# Off-Patent Transgenic Events: Challenges and Opportunities for New Actors and Markets in Agriculture

**DOI:** 10.3389/fbioe.2018.00071

**Published:** 2018-06-04

**Authors:** Patrick Rüdelsheim, Philippe Dumont, Georges Freyssinet, Ine Pertry, Marc Heijde

**Affiliations:** ^1^Perseus BVBA, Sint-Martens-Latem, Belgium; ^2^Association Française des Biotechnologies Végétales, Paris, France; ^3^Bio-EZ, Saint Cyr au Mont d'Or, France; ^4^Department of Plant Biotechnology and Bioinformatics, Ghent University, Gent, Belgium; ^5^International Plant Biotechnology Outreach, Vlaams Instituut voor Biotechnologie (VIB), Gent, Belgium

**Keywords:** off-patent event, generic, transgenic, GMO, data transportability, emerging markets

## Abstract

More than 20 years ago, the first genetically modified (GM) plants entered the seed market. The patents covering the first GM plants have begun to expire and these can now be considered as Off-Patent Events. Here we describe the challenges that will be faced by a Secondary Party by further use and development of these Off-Patent Events. Indeed, the conditions for Off-Patent Events are not available yet to form the basis for a new viable industry similar to the generic manufacturers of agrochemicals or pharmaceutical products, primarily because of (i) unharmonized global regulatory requirements for GM organisms, (ii) inaccessibility of regulatory submissions and data, and (iii) potential difficulties to obtain seeds and genetic material of the unique genotypes used to generate regulatory data. We propose certain adaptations by comparing what has been done in the agrochemical and pharmaceutical markets to facilitate the development of generics. Finally, we present opportunities that still exist for further development of Off-Patent Events in collaboration with Proprietary Regulatory Property Holders in emerging markets, provided (i) various countries approve these events without additional regulatory burdens (i.e., acceptance of the concept of data transportability), and (ii) local breeders agree to meet product stewardship requirements.

## Introduction

The first genetically modified (GM) plants were produced early in the 1980s by means of *Agrobacterium tumefaciens* as a vector to introduce a new gene into the plant as a trait of interest (Bevan et al., 1983; Herrera-Estrella et al., 1983). Numerous laboratories from the public and private sectors have worked on the production of GM plants, leading to the first commercial GM plants in the mid-1990s (James and Krattiger, 1996). Since then, many new GM crops have reached the market and been adopted all over the world. In 2016, more than 18 million farmers grew GM crops on a total of 185.1 million hectares in 26 countries, a 110-fold increase since the first releases (James, 2016), demonstrating the very successful adoption in global cropping systems despite intense societal debates. The main traits commercialized are herbicide tolerance and/or insect resistance

(James, 2016). More than 20 years after the initial commercialization, patents covering these profit-making Events have begun to expire. These patents were valid for 20 years after granting in the USA and Canada and after filing in other countries. In contrast to generic product development in the pharmaceutical and agrochemical industries, the current regulatory regimes for GM crops make it particularly challenging and currently virtually impossible to establish a viable generic industry in this sector.

The timeline for commercialization of an Event is long (~14 years for the first commercial launch) (Fraley, 2015) and the investment high (McDougall, 2011), in particular to comply with all the regulatory requirements, address the stewardship expectations, and assume the liabilities associated with GM crops, thereby reducing the market opportunities to only a few companies for limited crop/trait combinations.

Proprietary Regulatory Property (PRP) Holders must maintain regulatory approvals in the countries in which they intend to release Events for cultivation as well as in countries where plants containing the Event or the GM plant-derived products will be exported. In many countries, such approvals are limited in time and need to be renewed regularly. PRP Holders must also observe stewardship requirements and remain legally responsible for all issues related to product identity, quality, and performance. When the Event becomes off-patent, these requirements remain in force if the PRP Holder wishes to maintain the sales or if a Secondary Party wishes to commercialize the Event. Jefferson et al. (2015) provided insights into some of the challenges to be addressed in post-patent use of GM crops.

Here we discuss the difficulties faced by any potential Secondary Party who wishes to use or further develop these Off-Patent Events, among which are (i) lack of harmonization of the global regulatory requirements for GM crops, (ii) limited accessibility to regulatory submissions and data, and (iii) potential obstacles to obtain material of the unique Event upon which the regulatory dossier was created. Notwithstanding this problematic context, we present existing opportunities to further develop Off-Patent Event plants in collaboration with PRP Holders in new markets, provided that the concept of data transportability becomes widely accepted and that the product stewardship and the regulatory requirements are observed by all users at the global level.

We will not cover the generation of a Generic Event (see Glossary) that differs from an Off-Patent Event (see Glossary). The development of a Generic Event requires a complete regulatory package, even when some data on specific components of the Event can be obtained from the PRP Holder or are publicly available. Recently, an approach has been reported to produce generic glyphosate-tolerant soybean (*Glycine max*) (Rojas Arias et al., 2017). Regulatory and stewardship responsibilities for a Generic Event will be the same as for an Off-Patent Event and this will also be true for the liabilities that could be even more challenging for the developer of a Generic Event, which will probably be unpatentable.

## Off-patent events are not generic

Generic products are widespread in the pharmaceutical or agrochemical industries because of the specific legislation that facilitates their commercialization. In these agrochemical or pharmaceutical products, the off-patent active ingredient is a molecule or a protein (“biosimilar”) and is the same (or “similar” for a protein) as in the original product, even when the production process is different. In addition, the formulation of the active ingredient can have been modified (Alfonso-Cristancho et al., 2015). Although specific procedures were developed to facilitate the registration of generic or biosimilar products (such as possibilities for data bridging in regulatory applications and authorization to initiate a regulatory package of a product before expiration of the corresponding patent), generic products have to obtain their own commercial authorizations.

In the case of GM plants, the situation differs, because the intellectual property coverage does not protect a molecule or a protein, but an Event, and no legislation has yet been put in place in any country to facilitate the conditions for development, sale, and use of Off-Patent Events. Below, we will focus on the challenges to be faced by any Secondary Party wishing to further develop and use an Off-Patent Event.

## Intellectual property rights

An Event can be protected by several patents, covering, for instance, the DNA sequences used (promoter, coding sequence,…) to obtain the new trait, the technologies used to produce the Event, the Event itself, its use, and the specific detection tests used to identify its presence. Ten patents cover the soybean Event GTS-40-3-2 in the USA (Jefferson et al., 2015). When ascertaining that an Event is off-patent, one has to check the expiration of all the patents in the considered countries, i.e., countries for cultivation and for import, that cover the Event itself, its use, and its derived products. Indeed, one should take into account that the commercialized Event may not be patented as such, but any plant containing the construct for the trait and that, hence, the patent concerning the plant would also cover the commercial Event.

Even when an Event is off-patent, the commercial varieties derived from this Event may still be protected, either through a patent in the USA or through the Plant Variety Protection (PVP) Act in most other countries. In the USA, patented varieties cannot be used for breeding, whereas in Europe, for example, it is allowed to breed varieties under PVP. In this latter case, the derived varieties can be freely commercialized if the patented trait has been removed. However, if the trait is still patented, a license from the patent trait owner is necessary for as long as that patent is in force.

Let's assume that the Event and its derived varieties are completely off-patent, then the PRP Holder would be confronted by the situation in which unlicensed Secondary Parties could use the Off-Patent Event for breeding (for instance, to develop new varieties) and cultivation (for instance via farm-saved seeds). An unlicensed Secondary Party could possibly also utilize the Off-Patent Event to generate varieties with combined traits (i.e., “stacked” Events), but, in that case, the Secondary Party would require the necessary technical ability and possess the PRP-related information to fulfill the regulatory conditions for such stacked Events. Potential candidates would include seed companies experienced in developing and managing Events, public institutions capable of creating their own varieties under license from the PRP Holder (such as the University of Arkansas) (Miller, 2016), and individual farmers able to grow farm-saved seeds, provided again no previously signed technology use agreement exists with the PRP Holder that prohibits saving seeds for subsequent cultivation and that local legislation and licenses allow its application to purchased seed bags. In contrast, licensed seed companies are required to use the Event only in accordance with the terms of their license agreement that usually contain restrictions on its use, independently of the patent coverage, and generally, such restrictions survive the termination or expiration of the license agreement. In other words, an Off-Patent Event cannot be used in a manner that is not permitted by the license terms. The same terms would apply also to farmers who have signed a technology use agreement with the PRP Holder.

Thus, as the intellectual property rights of the Event and its derived varieties expire, the PRP Holder has to reconsider the value capture mechanisms and decide in due time on possible options: (i) continue the commercialization on its own and/or reach an agreement with Secondary Parties interested in the use of the Off-Patent Event, or (ii) discontinue sales and regulatory approvals. In this decision process, the market opportunity will be considered for stacked Events, in which the Off-Patent Event is combined via breeding with other Events, possibly still protected by intellectual property rights. Such combinations may allow novel applications of the Off-Patent Event.

Should a Secondary Party wish to develop, market, or use an Off-Patent Event independently from the PRP Holder, aspects related to the material, the regulatory requirements, and the stewardship should be taken into account.

## Representative plant material of the off-patent event

A Secondary Party interested in the use of an Off-Patent Event must first obtain legal access to the Event. If the Event itself has been patented, then seeds have generally been deposited in an International Depository Authority (IDA) under the Budapest treaty (WIPO, 2018), such as the American Type Culture Collection (ATCC) in the USA or the National Collections of Industrial, Food and Marine Bacteria (NCIMB) in the UK. After patent issuance, such deposited biological material must be made freely available to the public. The storage time in an IDA is at least 30 years (WIPO, 2002). However, a sample requested during the patent validity may not be used by the purchaser for any commercial use, because it would constitute a patent infringement. Moreover, under the ATCC Material Transfer Agreement, ATCC Material and Progeny “may only be used by the Purchaser's Investigator for research purposes and only in the Investigator's laboratory”—“Any commercial use of the Biological Material is strictly prohibited without the ATCC prior written consent” (Davis, 2011). Notwithstanding and independently of the ATCC restriction, under paragraph 5 of the Generic Event Marketability and Access Agreement (GEMAA), as amended on November 5 2015, PRP Holders agree to make the Event available to the GEMAA signatories (GEMAA, 2015).

## Regulatory strategy

At the time an Event becomes Off-Patent in the major agricultural markets, the PRP Holder will have developed a global data package and obtained approvals for commercialization in countries in which the GM crop is intended for cultivation and for export in countries in which the harvested Event-containing plants, parts or GM plant-derived products will be imported. In the case of the United States Department of Agriculture-Animal and Plant Health Inspection Service (USDA-APHIS), once an Event is deregulated, it is considered equivalent to any other free article without need for follow-up submissions, unless data emerge that significantly change the risk assessment. In contrast to the USDA, in many other countries such as China, the EU, and South Korea, approvals are limited in time and resubmissions must be scheduled to renew approvals and avoid costly disruptions of international commodity trade. A resubmission may be a formal request for extension, but most authorities require additional information accounting for the acquired knowledge and even updates of previous studies to meet redefined needs since the original approval. A third type of approval (such as the procedures of the United States Environmental Protection Agency [US EPA] for Plant Incorporated Protectants) is even more restrictive: similar to chemical crop protection products, it is conditional, i.e., an approval will have mandatory performance and reporting obligations, such as implementation of an insect resistance management plan, and is granted directly to one particular party. In addition, such an EPA approval may be provisional. The distinction between the different types of approvals is important when the consequences of the off-patent situation are evaluated.

Although the PRP Holder will probably not stop supporting the regulatory approvals for the Off-Patent Events abruptly, no continuation will be guaranteed, especially if the PRP Holder intends to replace the Off-Patent Event with an improved patented Event. Thus, to be able to develop, breed, or use the Off-Patent Event, any Secondary Party must ensure that the necessary permits are and remain in place.

For a USDA-APHIS deregulation, there is no need to request a second deregulation. For time-limited approvals, the Secondary Party should monitor whether the approvals have been, or are in the process of being, renewed by the Primary PRP Holder. When approvals expire in a given country, the Secondary Party will have to cease any unapproved use in that country or obtain new approvals. Alternatively, the Secondary Party could apply for its own authorizations, possibly the only option in administrative systems that provide party-dependent authorizations (such as the US EPA), but associated with high regulatory costs due to compilation, submission, and maintenance of the authorization and resulting in obligations for and liability of the Secondary Party.

In contrast to usually publicly available approvals, the PRP submissions are subject to confidentiality claims and are protected internationally under Article 21 of the Cartagena Protocol on Biosafety (Secretariat of the Convention on Biological Diversity, 2000) as well as possibly covered by copyright claims. The fact that some information is publicly available does not imply that it can be used to support a Second Party's own regulatory package or own application. For instance, in Europe, the regulatory system provides data protection to applicants, because Regulation 1829/2003/EU (Article 31) foresees that parties cannot use or refer to data submitted by the initial applicant in their application for 10 years, and under Directive 2001/18/EC (Article 25) the prohibition is unlimited in time.

In view of the difficulties for Secondary Parties to renew a particular approval (e.g., in China, South Korea, and the EU), it is very unlikely that Secondary Parties will have access to up-to-date information, because the data protection period starts from the submission date of specific information; in other words, Secondary Parties will not be allowed to use new information submitted as part of a resubmission until expiration of the protection period relevant for the renewal. However, if it is practically impossible and too expensive to establish its own complete safety package, a Secondary Party has always the possibility to negotiate access to submitted information with the PRP Holder.

## Safety data package

The GM Event safety is supported by a data package comprising studies explicitly providing information required by the decision makers. Whereas some information may be general, relevant to the trait (such as herbicide tolerance or insect resistance) or the gene (such as origin and nature of the nucleotide sequence and the corresponding protein) and be valid for several Events; most data, such as, for instance, the nucleotide sequence at the insertion site, the effect of the insertion on agronomical parameters, or the biological composition, are specific for each Event. In this case, it is important to demonstrate that the stud(y)(ies) has(ve) been conducted on the specific Event and molecular data and/or information on the genealogy of the material in support of the claims may be required by the authorities. The PRP Holder usually owns the study protocols and reports, and even when submitted as part of a data package, some level of protection may prevail or certain information may remain inaccessible due to confidentiality or copyright claims.

Whereas an initial data package serves to support market introduction, during the commercial lifetime of a GM crop additional data is accumulated and the data package is expanded. First of all, because the data package is submitted in various countries, the locally competent authorities may need specific data, requiring repetition of the initial study with an adapted study design or a completely new study. The PRP Holder can usually anticipate most requests for an acceptable study report but the problems expand when a country requires studies performed *in situ*. Secondly, over time, requirements change and are redefined, creating difficulties when a time-limited authorization expires and the authorities demand the submission of an up-to-date study design as part of the renewal. Finally, during large-scale implementation, unexpected findings might emerge that necessitate a specific effort to understand the discovery source and the impact on the risk assessment. In conclusion, the safety data package has to be substantially and continuously maintained throughout the life cycle of the Event, independently of its patent life. When a Secondary Party wants to independently engage in the use of an Off-Patent Event, a safety data package must be established as follows:

- by referring to the publicly available studies or data previously submitted by the PRP Holder, but usually not encompassing the entire safety package and, thus, rarely sufficient; indeed, the PRP Holder may have recently obtained information that has not been supplied to the competent authorities yet and, hence, are unknown and cannot be used by the Secondary Party;
- by agreeing with the PRP Holder on conditions for access to and use of the existing data;
- by establishing a proprietary data package by repeating the patented studies, requiring access to the biological material contained in the Off-Patent Event and legal permission to use such material for regulatory purposes. Under the current laws, at least in the USA, this data package can be initiated only when the Event or its constituents are off-patent.

## Compliance with approval conditions

An additional regulatory aspect relates to the conditions and liabilities associated with the approval. Depending on the type of Event and its approval, specific stipulations may be imposed. The PRP Holder is responsible for ensuring that all specifications are implemented, possibly by transferring part of its obligations to licensees, including farmers, via contracts and technology use agreements. For example, specific labeling of the (Event-containing) GM products may be mandatory to inform the farmers about the nature of the material or about particular management practices. In some cases, the implementation of an insect resistance management plan is a prerequisite for the approval. These examples illustrate that the regulatory obligations of the PRP Holder do not stop at the approval, but need to be maintained rigorously during the lifetime of the Event.

Upon patent expiry, the leverage of the PRP Holder over other users is in principle reduced. Facing continuous and onerous regulatory obligations, but less well equipped to impose conditions, the PRP Holder will re-evaluate whether to comply with the regulatory requirements. In addition, when Secondary Parties will supply the same material as a new source, there is a risk that they may not comply with all the regulations imposed on the PRP Holder. More importantly, in the case of non-compliance or any unexpected finding, the PRP Holder will be the first to be questioned and from whom liability and redress will be sought. Therefore, the incentives for a PRP Holder to discontinue sales and regulatory support for an Off-Patent Event and provide a new, patented Event as a substitute are extremely high.

## Product stewardship

Product Stewardship is the responsible management of a product from its launching through its use to its ultimate discontinuation. Although safety and compliance with legal obligations are inherent conditions to be observed, stewardship covers additional aspects of identity, purity, quality, and performance of GM crops and imposes a quality management system covering all Event handling that is subject to external audits. Furthermore, PRP Holders are expected to ensure the use of their products in a manner that respects the supply chain and does not disrupt international trade. The “Excellence Through Stewardship” (ETS) initiative was established by the biotechnology industry on a voluntary basis and promotes the adoption of stewardship programs and quality management systems across the full biotechnology plant product life cycle (www.excellencethroughstewardship.org). From this comprehensive stewardship program, some elements are particularly relevant for the discussion on Off-Patent Events. To avoid trade disruption, the developers (PRP holders) must ascertain that all required regulatory permits and authorizations are available in countries in which they intend to commercialize the Off-Patent Event and any derived products. For seed production, special care is taken to ensure the traceability and to avoid intermingling between non-GM and GM seed, as well as between different GM Events, requiring detailed knowledge of Event performance and characteristics, such as identity, genetic purity, and performance criteria. Along the value chain of the product, downstream users, i.e., farmers and downstream processing, need to be informed and trained for the optimal utilization of the Event, e.g., agricultural practices, labeling, channeling, and identity preservation. A specific case is the Integrated Pest Management (IPM) that aims at minimizing damage of pests, such as weeds, insects, and viruses, and maximizing the availability and longevity of the tools needed for the pest management. Irrespective of the impositions by the authorities, stewardship requires the developers to create their own IPM approach during the Research & Development phase, such as design of refuges of non-GM crops amidst GM insect-resistant crops. Due to the complexity of the process and the potential impact on multiple stakeholders, establishment of an Incident Response System is an essential part of any quality management system and is put in place as early as possible. Examples of incidents include improper functioning of the trait, an unintended, unauthorized release of the plant material in the environment, or a seed quality failure. Finally, developers must anticipate product discontinuation, as, for instance, when the commercial interest in a trait or particular Event has diminished and does not justify regulatory support continuation, implying a managing process to remove the specific Event from the market.

Any Secondary Party willing to further develop an Off-Patent Event will have to establish a stewardship program for the different development and commercialization steps of the Off-Patent Event. For practical purposes, in the USA, in view of the conditions imposed in article 13(a) of the GEMAA (GEMAA, 2015), the Secondary Party will have to become an ETS member and accept to be regularly externally audited. In addition, when such developments are done in collaboration with the PRP Holder, this PRP Holder may oblige the Secondary Party to have an audit system comparable to ETS.

## The precedent of the agaccord

To date, only the USA (through a voluntary, industry-negotiated agreement) established a framework agreement to manage Off-Patent Events, designated the AgAccord (www.agaccord.org). This framework comprises two separate agreements that cover the full spectrum of issues related to patent expiration: the GEMAA^SM^ (GEMAA, 2015) and the Data Use and Compensation Agreement (DUCA). The AgAccord supports business opportunities for parties seeking to use Off-Patent Events in the USA, while ensuring that all global regulatory commitments are maintained for Off-Patent Events and that the USA exports of the event-containing products are not disrupted. The AgAccord establishes a standard process to make available Off-Patent Events and the corresponding proprietary regulatory information otherwise not accessible to interested parties. In addition, this access begins prior to the patent expiration. However, the PRP Holder may choose to maintain all necessary authorizations and, thus, not exchange information or material. Although these agreements apply to the USA only, they constitute a starting point for the types of obligations that would be expected between a Secondary Party outside the USA and the PRP Holder, in particular in the area of stewardship.

## Market options and examples of off-patent utilization

In the case of the original glyphosate-tolerant Roundup Ready soybean Event, known as GTS 40-3-2, prior to the GEMAA instatement, Monsanto indicated its willingness to maintain full global regulatory support until 2021. Now that GEMAA is active, if Monsanto wants to discontinue the regulatory responsibilities for GTS 40-3-2, it needs to notify all interested parties at least 7 years prior to any such discontinuation. In such a notification, Monsanto, as PRP holder must set forth (i) the discontinuation date and (ii) whether it will retain or transfer the PRP ownership (GEMAA, 2015). In case of discontinuation, it has to announce the last sale. As Monsanto is commercializing a replacement Event for GTS 40-3-2, this Event will logically be discontinued in the future.

After patent expiration, new utilizations may be released for the Off-Patent Event, including for instance saving and replanting seeds of certain varieties in the fields by farmers, provided the originally purchased seeds are not covered by other patents or use restrictions in the seed bag license or in a technology use agreement. Since November 2014, such a use of GTS-40-3-2 has been possible: the University of Arkansas System Division of Agriculture released a glyphosate-tolerant soybean varieties UA 5414RR in December 2014 and UA 5715GT in April 2016, both available for sale to USA farmers without technology fees and without restrictions on farmer-saved seed (Miller, 2016). Thanks to a specific license from Monsanto, breeding material has been provided to public farmers, including the University of Arkansas (Miller, 2016).

In contrast, in April 2015, Event MON810 conferring insect resistance in corn (*Zea mays)* also became off-patent (GEMAA, 2013), but, since 30 September 2015, its approval by the US EPA as a corn product with a single plant-incorporated protectant has expired and, therefore, cannot be freely used by seed companies and farmers in the USA (U.S. Environmental Protection Agency, 2010). The plant-incorporated protectant in MON810, *Bacillus thuringiensis* Cry1Ab delta-endotoxin, retains an approved status for the US EPA. In this case, because the regulatory approvals for use as a single trait have not been maintained by the PRP Holder after the USA patent expiration, Secondary Parties in the USA have no direct opportunity to exploit the potential of the patent expiration without obtaining of a new permit. Following GTS-40-3-2 and MON810, a handful of Events will also probably become off-patent in the USA between 2014 and 2020, and several more after 2020.

These examples show that the possible use of Off-Patent Events without a large investment in the regulatory package remains very limited for Secondary Parties, because it strongly depends on agreements with the PRP Holders to have access to the Event and to keep approvals in force. Moreover, outside the USA, a contractual framework, such as the AgAccord that would facilitate possibilities for Secondary Parties, is lacking.

## Emerging market options and potential directions for further improvement

Many untapped opportunities remain for GM corn in emerging markets: “in Asia, there are about 60 million hectares of potential biotech maize, with 35 million hectares in China alone; there is a similar potential in Africa for up to 35 million hectares of biotech maize” (James, 2016). New GM corn markets in Africa will probably include Nigeria, Ethiopia, Namibia, Swaziland, and Malawi, and Vietnam in Asia. Secondary Parties will hopefully appear in such markets to create new plant breeding and commercial seed activities possibly in their own and the GM maize PRP Holder interests. In these countries as in many other African and South American countries, the patent status of an Event that has been commercialized elsewhere for 20 years is not an issue, because in most of them the Events have not been the subject of patent filings. Consequently, no intellectual property right for the Event exists in these countries and country-dependent patent rights are not extendable to countries where no filing has been done. In many countries, especially in Africa, the biosafety regulatory environment still needs full implementation. In addition, workable seed laws, variety certification procedures, and seed certification schemes are not regionally harmonized and effective, with negative outcomes for breeding investments and for the emergence of professionally certified seed production and reliable seed supplies.

Even if conditions existed favoring the emergence of local and professional seed companies, the African countries willing to regulate the cultivation of GM crops would need to accept the concept of data transportability to facilitate such a development: in agreement with the PRP Holder, data packages establishing the human and environmental safety of the Off-Patent Event agreed in experienced countries, such as South Africa, should be recognized as acceptable in other African countries. In this manner, risk assessment could be focused on studies that analyze the efficacy and environmental impact of the trait under local conditions. When countries have similar growing conditions and pests, data transportability can also apply to field data (Garcia-Alonso et al., 2014). With enhanced internationally harmonized regulatory systems, emerging markets may become the best place for non-conflicting collaborations between PRP Holders and Secondary Parties.

In addition, in the presence of a political willingness, the development of a generic industry for GM crops could be stimulated by initiatives, such as those developed in the pharmaceutical industry following the USA Drug Price Competition and Patent Term Restoration Act (Public Law 98-417). According to this research or safe harbor exemption, performing research and tests for the preparation of regulatory approvals does not constitute infringement for a limited term before the end of the patent term. This exemption allows manufacturers to prepare generic drugs in advance of the patent expiration. In the European Union, equivalent exemptions are allowed.

## Conclusion

Off-Patent Events for GM crops are and will increasingly become a reality, constituting a major challenge for PRP Holders. By maintaining authorizations, they remain responsible and liable for stewardship and have to keep data updated for regulatory compliance purposes, which is difficult when Secondary Parties use the Off-Patent Event. Although Off-Patent Events utilized as single Events might be scarce, they might be used in combinations with additional traits.

Currently, the GM crop regulatory systems do not facilitate a generic industry for Off-Patent Events. GM regulatory harmonization and simplification, including the acceptance of data transportability among countries and regions, would be a significant achievement for increased use and acceptance of the technology. Such improvements would allow a cost reduction and potentially open the market to new actors, in addition to the few multinationals that currently have the resources to develop and maintain GM Events.

Initiatives, such as the AgAccord are essential to facilitate the further development of Off-Patent Events. The founding members of the AgAccord could seemingly decide to extend the agreement territory to the rest of the world, without adverse impact on the members, but with many new opportunities for non-USA signatories. As mentioned above, improvements can also be made by taking advantage of the applications in the pharmaceutical industry to speed up the development of generic drugs.

Although emerging markets often still lack a regulatory environment that would allow the commercialization of GM crops, the most promising opportunities for Secondary Parties in direct collaboration with PRP Holders may reside in the African and Asian countries that are in the process of setting up a regulatory framework to handle GM crops for scientific research and for commercialization.

## Author contributions

PR, PD, GF, IP, and MH collected the information and wrote the paper.

### Conflict of interest statement

PR is owner and works for the company Perseus BVBA. GF is owner and chairman of the consultancy company Bio-EZ SARL. PD is an independent Board Member of the Association Française des Biotechnologies Végétales (AFBV) and of Calyxt, Inc. (New Brighton, MN, USA). The authors declare that the research was conducted in the absence of any commercial or financial relationships that could be construed as a potential conflict of interest.

## Glossary

*Event*: the unique recombinant DNA insertion event that took place in one plant cell, which was then used to generate a transgenic plant. The selected Event is used for breeding and development of commercial varieties in a crop.

*Generic*: as used here, refers to a product that is not protected by intellectual property rights and that is freely available for use by third parties for commercial and development purposes.

*Generic Event*: an Event that harbors the same inserted genetic sequences as the corresponding commercialized Event, but that is made *de novo* with nucleotide sequences and technologies available in the public domain.

*Off-Patent Event*: an existing Event, originally patented by, and commercially available through, a PRP Holder for which the patent protection has expired in a specific territory.

*Proprietary Regulatory Property* (PRP): The data, dossiers, and authorizations that enable the cultivation and sale of an Event in any countr(y)(ies) in which it is approved for cultivation and allow the importation and use of material containing that Event (seed product, grain, or any product thereof regulated as a result of the Event).

*Proprietary Regulatory Property (PRP) Holder*: an entity that owns or controls the PRP and any other relevant intellectual property rights for an Off-Patent Event.

*Secondary Party*: an entity that further develops or uses an Off-Patent Event.
